# Shiny Aging Murine Multi-Omic Analyzer: An Interactive Platform for Exploratory Multi-Omic Analysis of Murine Aging

**DOI:** 10.34133/csbj.0245

**Published:** 2026-11-10

**Authors:** Mayuka Ninomiya Kanda

**Affiliations:** Department of Bioengineering and QB3 Institute, University of California, Berkeley, Berkeley, CA 94720, USA.

## Abstract

Aging is accompanied by complex, tissue-specific molecular changes across multiple biological layers, yet the exploratory analysis of age-associated multi-omic datasets remains challenging for many experimental researchers due to technical and computational barriers. Here, I present Shiny Aging Murine Multi-Omic Analyzer (Shiny AMMOA), a graphical-user-interface-based, user-friendly analytical application that enables interactive exploration of curated murine aging-associated bulk transcriptomic, proteomic, and metabolomic datasets. Shiny AMMOA incorporates 3 predefined publicly available datasets within a unified R Shiny framework and supports end-to-end analyses, including differential expression testing, pathway enrichment analysis, and pathway-level visualization across individual omic layers and in cross-omic comparisons. Representative use cases illustrated how Shiny AMMOA can recapitulate key findings from original source studies, facilitate cross-omic comparison of pathway enrichment, and enable visualization of molecular changes across omics layers on Kyoto Encyclopedia of Genes and Genomes pathway diagrams. These use cases demonstrate the potential utility of Shiny AMMOA for exploring tissue-, pathway-, and modality-specific aging-associated alterations and for generating biologically informed hypotheses. By providing rapid access to curated large-scale public datasets through a user-friendly interface, Shiny AMMOA supports experimental biologists and aging researchers in exploring aging-associated datasets and generating hypotheses for further experimental investigation while reducing the need for intensive computational work. Shiny AMMOA is freely available at https://github.com/M-Ninomiya-Kanda/Shiny_AMMOA_local, and a lightweight web-based demonstration version with limited functionality is available at https://m-ninomiya-kanda-shiny-ammoa-web.share.connect.posit.cloud/.

## Introduction

Aging is a major risk factor for numerous chronic diseases, including cardiovascular, neurodegenerative, and metabolic disorders, and ultimately contributes to increased mortality [1,2]. Accordingly, understanding biomolecular changes associated with aging is of critical importance for identifying potential therapeutic strategies to promote healthy aging. Because aging affects multiple biological layers simultaneously [3,4], comprehensive characterization across multiple omics layers is often required to understand age-associated molecular alterations.

With advances in high-throughput technologies, multi-omic approaches are increasingly applied in aging research. However, generating such datasets from scratch requires substantial technical expertise and resources. To facilitate data reuse and open science, omics datasets are routinely deposited in public repositories upon publication [5]. However, availability of raw data does not necessarily translate into analytical accessibility. The degree of data curation of deposited omics datasets, including preprocessing, annotation, and formatting, varies substantially among studies, and many datasets require additional computational steps before downstream biological interpretation. These requirements can represent a substantial barrier for experimental scientists who may not be familiar with advanced statistical analyses.

Some studies have addressed this challenge by offering interactive, web-based data browsers that allow users to explore the specific omics datasets generated in their original studies [6–8]. While these tools facilitate intuitive exploration of individual expression patterns, they are typically designed for visualization purposes only and do not support advanced biostatistical analyses such as pathway enrichment or network-based investigation. Conversely, several graphical user interface (GUI)-based applications have been developed to enable sophisticated statistical analyses and visualization of omics data [9–11]. However, these tools generally require users to upload their own curated datasets, placing another burden of data preprocessing and quality control on the users’ end and creating an additional hurdle for noncoding experimental scientists. Therefore, these approaches still leave a gap between publicly available datasets and downstream biological interpretation. Together, these limitations highlight the need for an end-to-end, GUI-based workflow that connects curated public datasets with downstream statistical analysis and biological visualization.

At the level of biological interpretation, pathway enrichment analysis is a widely used approach for interpreting nontargeted omics datasets, including those generated in aging studies [12]. Although enrichment results can provide valuable insights into biological processes affected by aging, they often do not identify which specific molecules within a pathway contribute to the observed pathway alterations. Canonical pathway annotations typically encompass diverse functional components, such as extracellular signaling molecules, membrane receptors, cytosolic signaling mediators, and nuclear transcription factors, without explicitly accounting for their distinct roles or spatial organization [13,14]. Visualizing expression changes of all pathway components directly on pathway diagrams therefore complements enrichment analysis by facilitating interpretation of individual molecular alterations within their biological context [15].

Here, I present Shiny Aging Murine Multi-Omic Analyzer (Shiny AMMOA), a GUI-based application developed using the R Shiny framework. Shiny AMMOA provides access to 3 predefined and preprocessed public datasets spanning 3 omic layers (bulk RNA sequencing [RNA-seq], proteomics, and metabolomics) and multiple tissues in *Mus musculus*. Shiny AMMOA supports exploratory comparison across individual omic layers rather than formal integrative multi-omic analysis. By combining differential expression analysis, pathway enrichment analysis, and pathway-level visualization of molecular changes, Shiny AMMOA provides an end-to-end workflow for exploring age-associated molecular changes across omic layers and supports experimental scientists in generating biologically informed hypotheses for further investigation without requiring extensive computational expertise.

## Materials and Methods

### Data source

The Tabula Muris Senis bulk RNA-seq dataset [6] was selected as the reference transcriptomic dataset for Shiny AMMOA because of its broad tissue coverage and established use in studies of transcriptional aging. To identify comparable datasets for the other omic layers, publicly available age-related bulk omics datasets with broad multi-tissue coverage were searched. Among the studies available in peer-reviewed publications, including articles published online but not yet assigned to a journal issue, as of 2025 October 20, 2 datasets generated using C57BL/6-derived mouse strains and covering multiple tissues were selected as the proteomic and metabolomic sources for Shiny AMMOA.

The 3 source datasets were retrieved from publicly available sources associated with the original publications. Specifically, bulk RNA-seq data [6] were obtained from the Gene Expression Omnibus under accession number GSE132040, proteomic data [16] were retrieved from FigShare (DOI: 10.6084/m9.figshare.19765849), and metabolomic data [7] were extracted from the supplementary table of the original publication.

The main characteristics of the 3 source datasets are summarized below and Table S1, with further details available in the corresponding original studies. The effective sample size of tissue samples for each omic layer, tissue, age group, and sex group is summarized in Table S2.

The bulk transcriptomic dataset was derived from the Tabula Muris Senis bulk RNA-seq resource, a comprehensive murine aging atlas spanning multiple tissues and age groups. Bulk RNA-seq profiles were obtained from male and female C57BL/6JN mice across early adulthood to advanced age, with samples collected from 17 distinct tissues. Proteomic data were obtained from a discovery-based tandem mass tag proteomics study characterizing age- and sex-associated proteomic changes across murine tissues. This dataset comprises quantitative proteome measurements from 10 tissues collected from C57BL/6J mice at 8 and 18 months of age, including both male and female animals. Metabolomic data were sourced from a comprehensive study investigating age-related metabolic alterations in mice. The dataset includes metabolite abundance profiles from 15 tissues, including serum, obtained from young (3 to 6 mo) and aged (≥21 mo) C57BL/6J mice of both sexes. Metabolites were quantified using hydrophilic interaction chromatography coupled with high-resolution quadrupole–Orbitrap mass spectrometry.

### Bulk RNA-seq data preprocessing

Raw bulk RNA-seq count data were downloaded from the Gene Expression Omnibus under accession number GSE132040. Using the same quality filtering criteria as applied in the original publication [6], samples with a total read count below 4 million were excluded from subsequent analyses. The feature-level rows corresponding to read-count assignment categories (i.e., “__no_feature”, “__ambiguous”, “__too_low_aQual”, “__not_aligned”, and “__alignment_not_unique”) were removed and were not included as biological gene features in downstream DESeq2 analyses. The raw count data matrix was analyzed separately for each tissue using the standard DESeq2 pipeline [17] (version 1.52.0), with sex and age included as covariates in the design formula. In differential expression analyses comparing 2 age groups (e.g., 3 mo vs. 21 mo), age was treated as a binary variable. In contrast, for analyses including all age groups (i.e., 1, 3, 6, 9, 12, 15, 18, 21, 24, and 27 mo), age was modeled as a continuous variable after scaling. In the all-age-group analysis, the scaling was defined such that a log2 fold change of 1 corresponds to a doubling of mean expression over a 12-month interval.

Sex was included as a covariate in all-age-group analyses and in 2-group comparisons in which both age groups included male and female samples. These analyses used the following design formula:

expression∼sex+age_month (1)

In comparisons in which one or both age groups included only male samples (typically comparisons involving the 24- or 27-mo age groups), sex was not included as a covariate, and the following design formula was used:

expression∼age_month (2)

Gene-level filtering was performed according to the default behavior of DESeq2. Differential expression results were reported as log_2_ fold changes after shrinkage using the apeglm algorithm [18], as implemented in DESeq2. Before volcano-plot visualization and pathway enrichment analysis, genes that could not be mapped to an Entrez Gene ID were removed. Gene identifiers were annotated using AnnotationDbi (version 1.74.0) and org.Mm.eg.db (version 3.23.0).

### Proteomic data preprocessing

For differential expression analyses of the proteomic data, intermediate data files generated by the authors of the original publication [16] were retrieved from FigShare (DOI: 10.6084/m9.figshare.19765849) and used in Shiny AMMOA. Briefly, in the original publication, protein abundance estimates were obtained following batch-aware normalization and ratio scaling and were represented on a log_2_ scale. Subsequently, abundance values were modeled by the original authors using ordinary least squares (OLS) regression, with age, sex, and analytical batch included as covariates. Under this modeling framework, the estimated age coefficients correspond to covariate-adjusted log_2_ fold changes between young (8-mo-old) and aged (18-mo-old) mice. Accordingly, these values are labeled as “covariate-adjusted Log2FC” in the Shiny AMMOA application. Ensembl gene IDs, official gene symbols, estimated age coefficients (corresponding to covariate-adjusted log_2_ fold changes), and *P* values were directly retrieved from the intermediate data files generated by the original authors. For the preprocessed dataset used in Shiny AMMOA, false discovery rate (FDR) values were subsequently calculated using the Benjamini–Hochberg method. Ensembl gene IDs were mapped to Entrez gene IDs using the AnnotationDbi package (version 1.74.0) and the org.Mm.eg.db package (version 3.23.0). When multiple Entrez gene IDs were mapped to a single Ensembl gene ID, the Entrez gene ID with the smallest numerical identifier was retained, and the other mappings were excluded.

### Metabolomic data preprocessing

Metabolite abundance data were obtained as processed ion count data from the supplementary data table of the original publication [7]. For the preprocessed dataset used in Shiny AMMOA, differential-abundance analysis was performed using OLS regression, with log_2_-transformed ion counts as the response variable and age and sex included as covariates. In this modeling framework, the estimated age coefficients represent covariate-adjusted log_2_ fold changes between young and aged mice. Unlike the original study, interaction terms were not included in the OLS model in order to maintain consistent covariate adjustment across omics layers such as the transcriptome and proteome. The impact of omitting interaction terms was evaluated by comparing the resulting estimates with those reported in the original study, which showed comparable results (Supplementary Appendix 1).

Metabolite common names were mapped to Kyoto Encyclopedia of Genes and Genomes (KEGG) and Human Metabolome Database (HMDB) identifiers using MetaboAnalyst 6.0 (web version) [19], with manual curation. All MetaboAnalyst ID conversion queries described below were performed in October 2025. In the first round, 426 unique compound names annotated by the authors of the original source study were submitted to the “Metabolite ID Conversion” tool in MetaboAnalyst 6.0. Of these, 316 (74%) were matched, including 266 (62%) with KEGG IDs and 311 (73%) with HMDB IDs. Among the 110 compound names that were not matched in the first round, 95 were manually curated by assigning alternative common names identified from public databases such as HMDB and PubChem and were subsequently resubmitted to MetaboAnalyst 6.0. In the second round, 80 (84% of the 95 resubmitted compounds) were successfully matched, including 34 (36%) with KEGG IDs and 65 (68%) with HMDB IDs. Overall, 300 (70%) of the original 426 compound names were mapped to KEGG IDs and 378 (88%) to HMDB IDs after the 2 rounds of matching. The detailed queries submitted in the first and second rounds, including the manually revised compound names, together with the corresponding MetaboAnalyst responses, are provided in Table S3. Compounds that remained unmatched or lacked corresponding KEGG or HMDB identifiers were retained for visualization in the volcano plots but excluded from the corresponding enrichment analyses (see “Enrichment analysis” section).

### Enrichment analysis

For transcriptomic and proteomic data, Shiny AMMOA supports 2 widely used enrichment analysis algorithms: over-representation analysis (ORA) and gene set enrichment analysis (GSEA). In contrast, only ORA is supported for metabolomic data. GSEA was not implemented for metabolomic data because the limited number and nature of the analytes make this approach less informative in this context: 300 metabolites were mapped to KEGG identifiers for KEGG-based enrichment analysis, and 378 metabolites were mapped to HMDB identifiers for SMPDB-based enrichment analysis (see “Metabolomic data preprocessing” section).

In the local version of Shiny AMMOA, ORA is performed on demand in response to user-specified input. For transcriptomic and proteomic data, ORA querying KEGG and Gene Ontology (GO) pathways is conducted using clusterProfiler (version ≥ 4.20.0). For metabolomic data, ORA querying KEGG pathways is performed using enricher() function in the clusterProfiler package, using a compound-to-term annotation table compiled in September 2025 and stored as part of the application data. In contrast, ORA querying the Small Molecule Pathway Database (SMPDB) is performed using enrichHMDB() function in MicrobiomeProfiler package (version ≥ 1.18.1), which operates as an extension of and depends on clusterProfiler. The default thresholds were selected based on commonly used cutoff values and the distribution of differential expression statistics in each omic layer. For transcriptomic data, the default thresholds were set to FDR <0.05 and |log_2_ fold change| >0.5, whereas for proteomic and metabolomic data, the default thresholds were set to FDR <0.1 and |log2 fold change| >0. Differentially expressed genes, proteins, or metabolites are defined according to user-specified thresholds for FDR and log_2_ fold change and subsequently submitted to the corresponding enrichment analyses.

For transcriptomic and proteomic data, all measured genes or proteins successfully annotated with Entrez gene IDs were used as the background universe for ORA. For metabolomic data, the default background universe was defined as all measured metabolites in the user-specified tissue with successful KEGG ID annotation for KEGG enrichment analysis and HMDB ID annotation for SMPDB enrichment analysis. Given the limited compound coverage of the metabolomic datasets, which may substantially reduce the power to detect significantly enriched pathways, users can also select the “All metabolites registered in DB” option. When this option is selected, all metabolites registered in the respective KEGG or SMPDB database are used as the background universe. A caution message is displayed to users: “‘All metabolites registered in DB’ uses all KEGG/SMPDB metabolites; it may detect more pathways but can increase false positives.”

In contrast to ORA, GSEA is precomputed, and Shiny AMMOA displays the stored results. This architecture was adopted because GSEA generally requires greater memory resources and longer computation times than ORA, making on-demand computation less suitable for a GUI-based platform. In addition, GSEA does not depend on user-defined differential expression thresholds, making its results amenable to pre-computation and storage. For transcriptomic data, GSEA is available only for analyses across all age groups because precomputing GSEA results for every possible pairwise age-group comparison among 1, 3, 6, 9, 12, 15, 18, 21, 24, and 27 months would substantially increase the storage requirements. All measured genes or proteins successfully annotated with Entrez gene IDs were ranked in descending order according to their covariate-adjusted log_2_ fold-change values and subjected to GSEA using the default parameters of the gseKEGG() and gseGO() functions in clusterProfiler (version 4.20.0). Because the GSEA algorithm internally uses random numbers, a fixed random seed was used to ensure reproducibility.

In the web version of Shiny AMMOA, both ORA- and GSEA-based enrichment analysis results were precomputed and stored as in-app data (see “Lightweight web version development” section).

Enrichment results are visualized as dot plots. For transcriptomic and proteomic analyses, when more than 10 significant (adjusted *P* value < 0.05) pathways are identified, pathways are grouped into 5 clusters based on the similarity of their component genes or proteins. Semantic similarity is computed using enrichplot (version ≥ 1.32.0), and clustering is performed using Ward’s D2 hierarchical clustering algorithm implemented in stats (version ≥ 4.6.0). When more than 25 significant pathways are detected, only the top 5 pathways from each cluster, ranked by adjusted *P* value, are displayed to reduce redundancy.

### KEGG pathway mapping

On the “Pathway Mapping” page, age-associated changes in gene, protein, and metabolite expression are visualized using the pathview package [15] (version ≥ 1.52.0). Pathview is an R/Bioconductor package that maps user-provided expression data onto KEGG pathway diagrams using a heat color scale, enabling intuitive visualization of the locations of up- and down-regulated molecules within each pathway. In Shiny AMMOA, rectangular nodes represent genes or proteins, whereas small circular nodes represent metabolites. Differential expression results, represented as log_2_ fold-change values, are passed to the pathview() function, and the resulting heat-colored pathway diagrams are displayed in the user interface.

### KEGG licensing consideration

Shiny AMMOA, both fully functional local version and web-based demonstration version, was implemented with consideration of the licensing conditions specified in the KEGG Copyright and Disclaimer (https://www.kegg.jp/kegg/legal.html, last accessed 2026 Sep 10). Shiny AMMOA uses the pathview package [15] to generate expression-data-overlaid KEGG pathway maps in the local version of the application. KEGG-derived pathway images and pathway data are not distributed with the Shiny AMMOA. Instead, when a user requests a KEGG pathway visualization in the local application, the required KEGG pathway resources are retrieved from KEGG by pathview during execution and processed within the user’s local environment. The resulting visualization is therefore generated on demand rather than being redistributed by Shiny AMMOA. The web version of Shiny AMMOA does not display KEGG pathway maps. This restriction was implemented to avoid providing KEGG-derived pathway content as part of a hosted web service and reflects the licensing conditions governing the use of KEGG. The KEGG Copyright and Disclaimer states that academic use for providing services is subject to an academic service provider license.

Importantly, the licensing of the Shiny AMMOA software is independent of the licensing conditions applicable to KEGG content. Even when using the local version, users are responsible for ensuring that their use complies with the terms and conditions specified by KEGG. In particular, nonacademic users should obtain the appropriate KEGG license before using KEGG data or pathway maps. To reduce the risk of misuse, Shiny AMMOA displays a notice reminding users of the KEGG licensing conditions in the pathway visualization tab.

### R Shiny application development and implementation

Shiny AMMOA was implemented as a GUI-based application using the R Shiny framework to enable intuitive exploration of 3 predefined datasets across 3 omic layers. A comparison of Shiny AMMOA with other existing GUI-based tools is provided in Table S4. The overall structure of the application follows the schematic workflow shown in Fig. 1. First, omics datasets and associated analysis results are retrieved from publicly available repositories (see Source Data section). Second, these datasets undergo preprocessing steps specific to each omics layer, including data curation and calculation of differential expression values (see the corresponding Data Preprocess sections), and are stored as application-ready data objects. Third, selected analyses are performed dynamically within the application in response to user input. Within this workflow, users specify a set of analysis parameters, such as the data type (bulk RNA-seq, proteome, or metabolome), tissue of interest, and thresholds for differential expression and pathway enrichment. Based on these inputs, the application executes on-demand analyses, including pathway enrichment and pathway visualization, and displays the results through interactive plots and tables. Internet access is required to run Shiny AMMOA because certain analyses access external resources during execution.

**Fig. 1.**
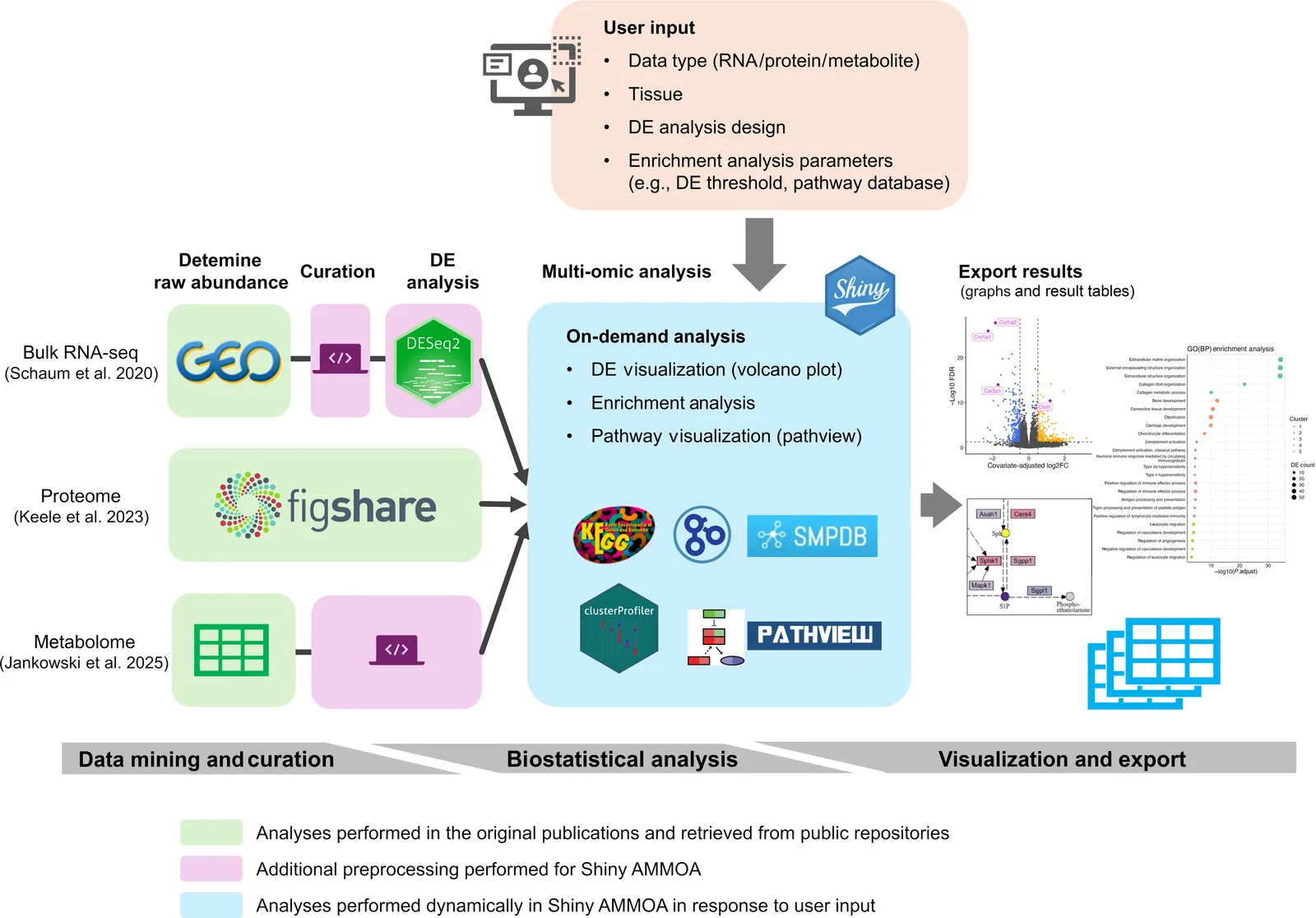
Workflow of Shiny Aging Murine Multi-Omic Analyzer (AMMOA) implementation. Three omics datasets (bulk RNA-seq, proteomics, and metabolomics) were retrieved from public repositories (see “Materials and Methods” section) in either raw or curated form, followed by additional data curation and differential expression analysis when required. Downstream analyses, including pathway enrichment, pathway visualization, and graph rendering, are performed dynamically in response to user input in Shiny application. Analytical results can be downloaded as figures (PNG format) and result tables (CSV format). DE, differential expression; FC, fold change; FDR, false discovery rate; GO(BP), Gene Ontology Biological Process.

### Lightweight web version development

The web version of Shiny AMMOA was developed solely to provide prospective users with a lightweight demonstration of the user interface and analytical workflow prior to local installation. The web version is deployed on the Posit Connect Cloud platform (Posit Software, PBC; https://connect.posit.cloud). A feature-by-feature comparison is provided in Table S5. Due to external database terms and conditions, as well as resource limitations such as server memory and CPU capacity of Posit Connect Cloud, several features available in the local version are restricted in the web version. In consideration of external database policies, KEGG pathway diagrams are not displayed because of licensing restrictions (see “KEGG licensing consideration” section). To reduce computational demands on the web server, all enrichment analysis results, including ORA, in the web version are precomputed rather than calculated dynamically in response to user input. Consequently, the web version provides more limited flexibility in user-specified parameters and supported age-group comparisons than the local version. For example, in the web version, the threshold parameters used to define differential expression (FDR and log_2_ fold change) cannot be adjusted, and transcriptomic analyses are limited to comparisons across all age groups or between 3 and 18 months.

### Software

All omics data preprocessing was performed in R (version 4.6.0). The local version of Shiny AMMOA was developed and tested using R (version 4.6.0), RStudio (version 2026.08.2+200), and the Shiny package (version 1.13.0). Package names and version information for individual analyses are provided in the corresponding sections above and summarized in Table S6.

## Results

### Architecture of the app

Shiny AMMOA is designed to facilitate interactive analysis and visualization of age-associated molecular changes in 3 predefined datasets across 3 omics layers without requiring direct programming by the user. A simplified workflow of Shiny AMMOA development is illustrated in Fig. 1. The application supports analyses across 3 omics layers: bulk transcriptomics, proteomics, and metabolomics. Source datasets for each omics layer were retrieved from publicly available repositories (see “Materials and Methods” section). As of version 2.0, Shiny AMMOA consists of 6 tabs (Fig. 2, screen captures): “bulk RNA”, “Proteome”, “Metabolome”, “Pathway Mapping”, “User Guide”, and “About”. In the “bulk RNA”, “Proteome”, and “Metabolome” tabs, users can perform differential expression analysis and pathway enrichment analysis for individual omics layers. Additionally, the “Pathway Mapping” tab enables users to generate heatmap-colored KEGG pathway diagrams that simultaneously display molecular changes derived from independent source datasets across the 3 omic layers.

**Fig. 2.**
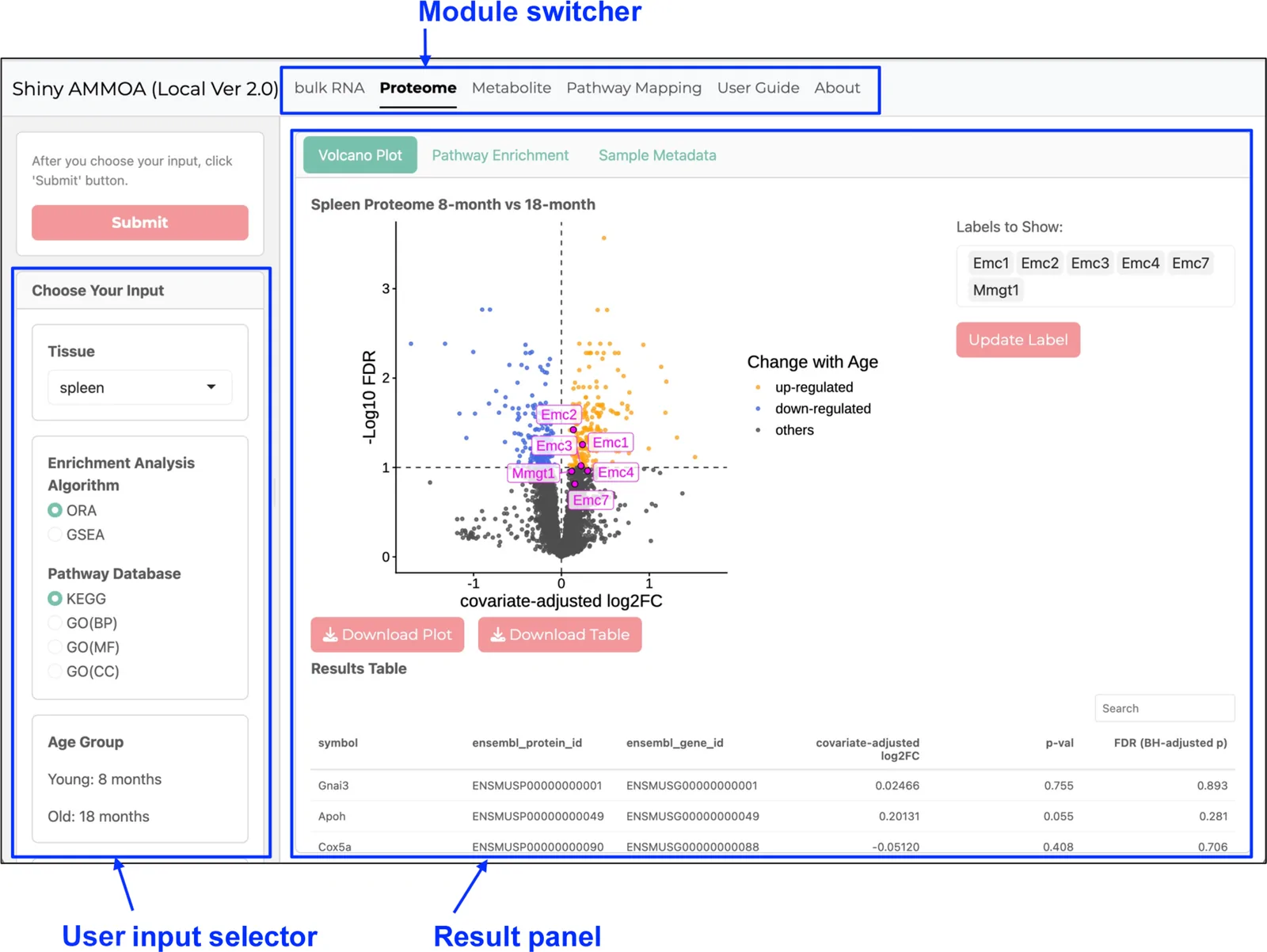
User interface of Shiny AMMOA. Upper panel: module switcher for selecting analytical modules or accessing the user guide. Left panel: user input controls. Main panel: display area for analytical results.

Shiny AMMOA is primarily designed to run in a local environment (i.e., on the user’s own computer). To allow prospective users to explore the graphical user interface and overall workflow before installation, a lightweight web-based demonstration version is also available at https://m-ninomiya-kanda-shiny-ammoa-web.share.connect.posit.cloud/. This demonstration version is intended solely to showcase the interface and workflow and does not provide the full analytical capabilities of the local version. Details of the implementation and functional limitations of the web-based version are described in the “Materials and Methods” section and Table S5.

### Validation of Shiny AMMOA

To quantitatively assess whether Shiny AMMOA can reproduce findings reported in the original source publications, I compared results generated by Shiny AMMOA with those reported in the corresponding original studies. Two levels of results were considered for validation where comparable results were available: differential expression and functional enrichment. Detailed validation procedures and results are provided in Supplementary Appendix 1.

Briefly, for the proteomic layer, the differential expression results used in Shiny AMMOA were obtained from the same intermediate results generated by the original study and therefore were not independently validated. Comparison of functional enrichment results showed substantial agreement, with 76% to 86% of significantly enriched GO Biological Process [GO(BP)] terms identified by Shiny AMMOA overlapping with those reported in the original study, although Shiny AMMOA generally yielded a more conservative set of enriched terms, potentially reflecting differences in analytical and annotation settings between the 2 analyses, including differences in enrichment cutoff criteria. For the metabolomic layer, age-associated log_2_ fold-change estimates generated by Shiny AMMOA were highly concordant with those reported in the original study across all 15 tissues, with Spearman correlation (rho) ranging from 0.941 to 1.000. Functional enrichment analysis was not performed in the original study and therefore could not be evaluated. Separately, because the current metabolomic workflow uses an additive age + sex model (see “Metabolomic data preprocessing” section), a sensitivity analysis was performed to assess the effect of including an age × sex interaction. The 2 model specifications identified different sets of metabolites passing the default FDR <0.1 threshold, with Jaccard indices ranging from 0.233 to 0.757 across tissues. These findings indicate that metabolomic differential-abundance and ORA results should be interpreted as model-dependent exploratory findings. For the transcriptomic layer, quantitative validation against the original study was not feasible because publicly available resources did not provide downloadable differential expression results or directly comparable tissue-specific enrichment results.

### Use case 1: exploring single-omic analysis

A representative output of the “Proteome” module is shown in Fig. 3A and B. To assess reproducibility relative to the original source study, age-associated changes in the spleen proteome were visualized (Fig. 3A), followed by GO(BP) enrichment analysis of proteins increased with age (Fig. 3B). Shiny AMMOA recapitulated key findings of the original publication, including an age-associated increase in endoplasmic-reticulum-related proteins in the spleen [16]. Minor differences between the original publication and Shiny AMMOA outputs are likely attributable to differences in analytical settings, such as pathway redundancy handling and gene set size thresholds used in enrichment testing.

**Fig. 3.**
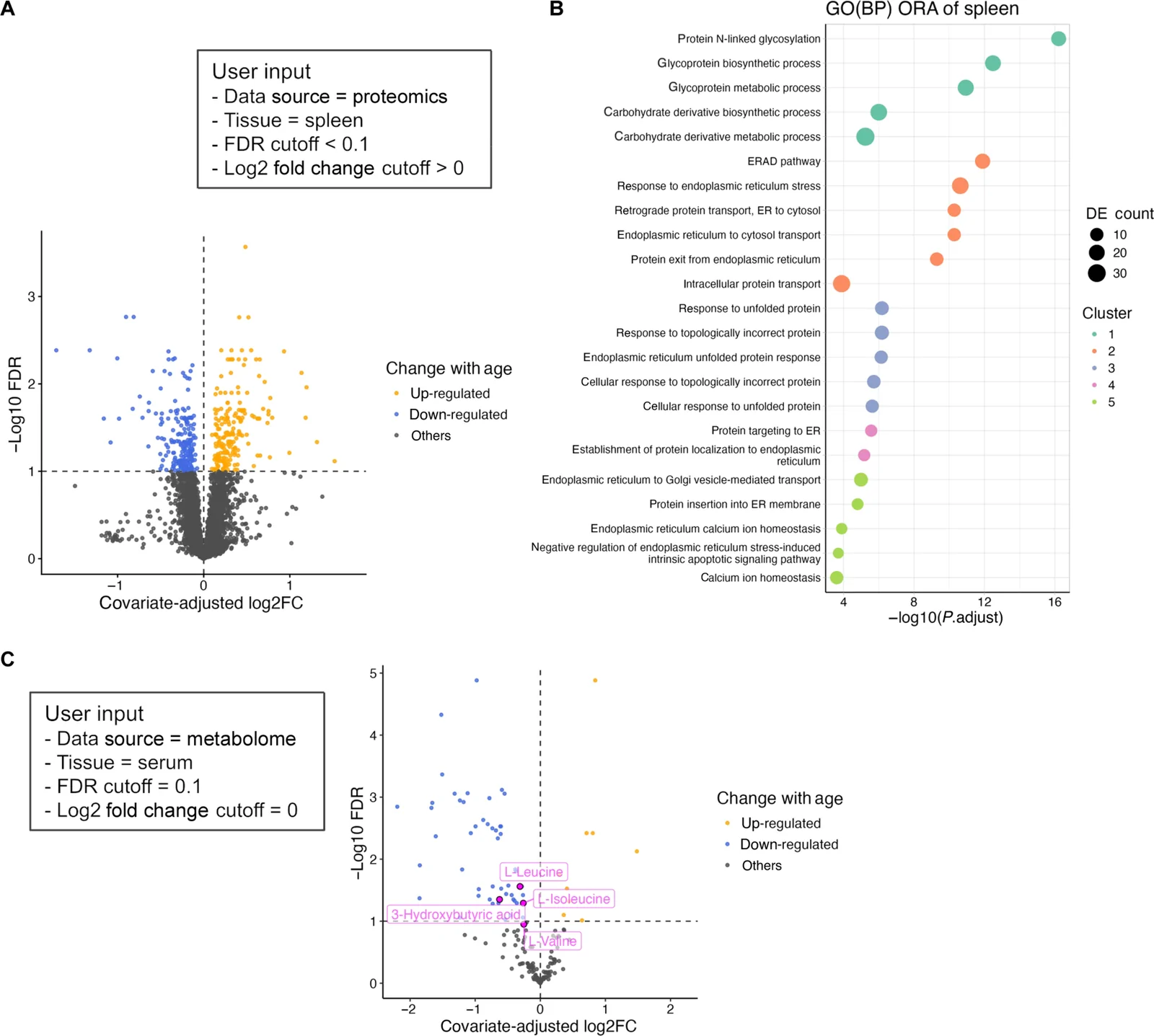
Representative outputs of the enrichment analysis modules in Shiny AMMOA. (A) Volcano plot showing age-associated proteomic changes in murine spleen (8 mo vs. 18 mo). Proteins exceeding user-defined DE thresholds (FDR < 0.1, |log_2_ fold change| > 0) are highlighted. (B) GO(BP) enrichment results derived from proteins increased with age in (A). Significantly enriched biological terms are clustered based on gene-set similarity, and the top 5 terms from each cluster are displayed (see “Materials and Methods” section). ORA, over-representation analysis. ERAD, endoplasmic reticulum-associated degradation; ER, endoplasmic reticulum. (C) Volcano plot showing age-associated changes in circulating metabolites in serum samples (3 to 6 mo vs. >21 mo). Metabolites exceeding user-defined DE thresholds (FDR < 0.1, |log_2_ fold change| > 0) are highlighted.

As enrichment analysis results vary depending on analytical parameters, it is sometimes recommended to perform sensitivity analyses by applying various sets of analytical parameters. The local version of Shiny AMMOA enables interactive adjustment of enrichment analysis parameters, including the analytical algorithm (ORA or GSEA) and differential expression thresholds. Figure S1 demonstrates how enrichment analysis results vary with different parameter settings using spleen bulk RNA-seq data. As expected, a stringent differential expression threshold (FDR < 0.05 and |log_2_ fold change| > 2) resulted in no GO terms being significantly enriched, likely due to the very limited number of genes meeting the differential expression criteria, whereas more relaxed thresholds resulted in more significantly enriched GO terms. Nevertheless, several GO terms, such as “B cell mediated immunity” (GO:0019724), appeared under multiple parameter settings, suggesting the robustness of these observations. This finding is potentially consistent with previously reported age-associated changes in immune cell composition and function [20,21].

To further illustrate the utility of this single-omic exploration feature, a representative result of the “Metabolome” module is shown in Fig. 3C. Consistent with the original publication [7] and other independent prior studies [22,23], serum levels of 3-hydroxybutyrate, a major circulating ketone body, were significantly decreased with age. Several amino acids, including the branched-chain amino acids L-leucine and L-isoleucine, also showed age-associated decreases as described in the source publication, whereas L-valine exhibited a similar trend but with FDR ≥0.1. Notably, tissue-level analyses revealed that 3-hydroxybutyrate was reduced with age in liver and quadriceps muscle but not in soleus muscle (Fig. S2), suggesting tissue-specific age-associated alterations in 3-hydroxybutyrate abundance, with more pronounced decreases in liver and glycolytic muscle than in soleus muscle.

### Use case 2: cross-omic comparison of pathway enrichment

mRNA and protein abundance may be regulated by distinct biological processes, and age-associated changes at the transcript and protein levels do not necessarily concord. Indeed, in the source article of the proteomic dataset used in Shiny AMMOA, Keele *et al*. [16] compared age-associated effects estimated from protein and transcript levels using the same bulk RNA-seq data incorporated into Shiny AMMOA and reported correlation coefficients of 0.20 and 0.04 in the kidney and heart, respectively. Another study analyzing both mRNA and proteomic profiles from the same mouse samples similarly reported a modest correlation between age-associated effects at the transcript and protein levels (*r* = 0.235) [24]. Nevertheless, an important question is whether age-associated alterations at the transcript and protein levels show overlap at the pathway level, even when individual genes or proteins do not show strong concordance. Although preliminary, Shiny AMMOA provides a simple and rapid way to examine this question by allowing a similar analytical workflow to be readily applied separately to independent transcriptomic and proteomic datasets, facilitating comparison of pathway-level results across omics layers. Figure 4 shows GO(BP) enrichment of genes and proteins down-regulated with age (FDR < 0.1 and log_2_ fold change < 0) in bulk RNA-seq (Fig. 4A) and proteomic (Fig. 4B) analyses, respectively. Interestingly, pathways related to lipid metabolism, such as “fatty acid catabolic process” (GO:0009062), were enriched in both the transcriptomic and proteomic analyses. Previous studies have implicated impaired fatty acid oxidation in age-associated renal fibrosis and kidney disease [25,26]. The shared enrichment of fatty acid catabolic processes in both transcriptomic and proteomic analyses is therefore consistent with previously reported age-associated alterations in renal fatty acid metabolism. Together, this example illustrates how Shiny AMMOA can facilitate the identification of biological processes showing concordant age-associated changes across independent omics datasets and may support hypothesis generation for further investigation.

**Fig. 4.**
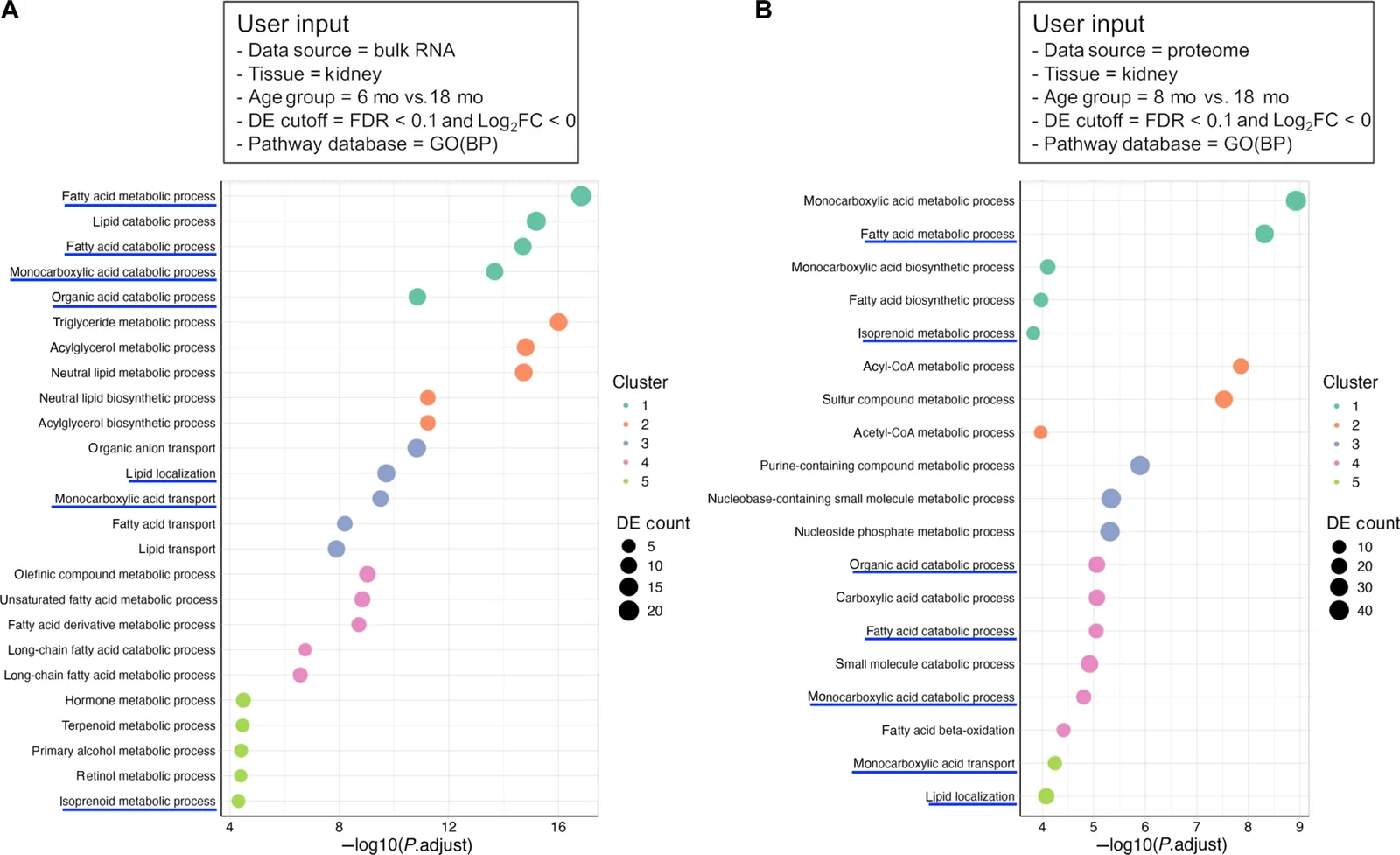
Representative output of cross-omic comparison in Shiny AMMOA. (A) GO(BP) enrichment results derived from transcripts decreased with age in murine kidney (6 mo vs. 18 mo). Genes meeting user-defined differential expression thresholds (FDR < 0.1, log_2_ fold change < 0) are used as foreground genes. (B) GO(BP) enrichment results from the proteomic dataset using comparable user-defined parameter settings as in (A), but for a different source dataset and age comparison (8 mo vs. 18 mo). GO(BP) terms identified in both transcriptomic and proteomic analyses are highlighted with blue underlines.

### Use case 3: pathway diagram visualization

Figure 5A visualizes age-associated transcriptomic changes of genes involved in the “ECM–receptor interaction” pathway (KEGG ID: mmu04512) in limb muscle (tibialis anterior). Consistent with the source data article [6] and other independent studies [27–29], many pathway genes exhibited significant age-dependent alterations, potentially reflecting dysregulated extracellular matrix (ECM) remodeling during aging [27]. Importantly, visualization of individual pathway components on the KEGG diagram revealed that expression changes were not uniform across functional categories: While most ECM structural components, such as collagens, showed a monotonic decline with age, integrin genes displayed more subtype-specific patterns. This distinction is biologically informative, as integrins primarily function as ECM receptors rather than structural components [30], highlighting how pathway visualization can facilitate intuitive interpretation of functionally heterogeneous aging patterns within a single pathway.

**Fig. 5.**
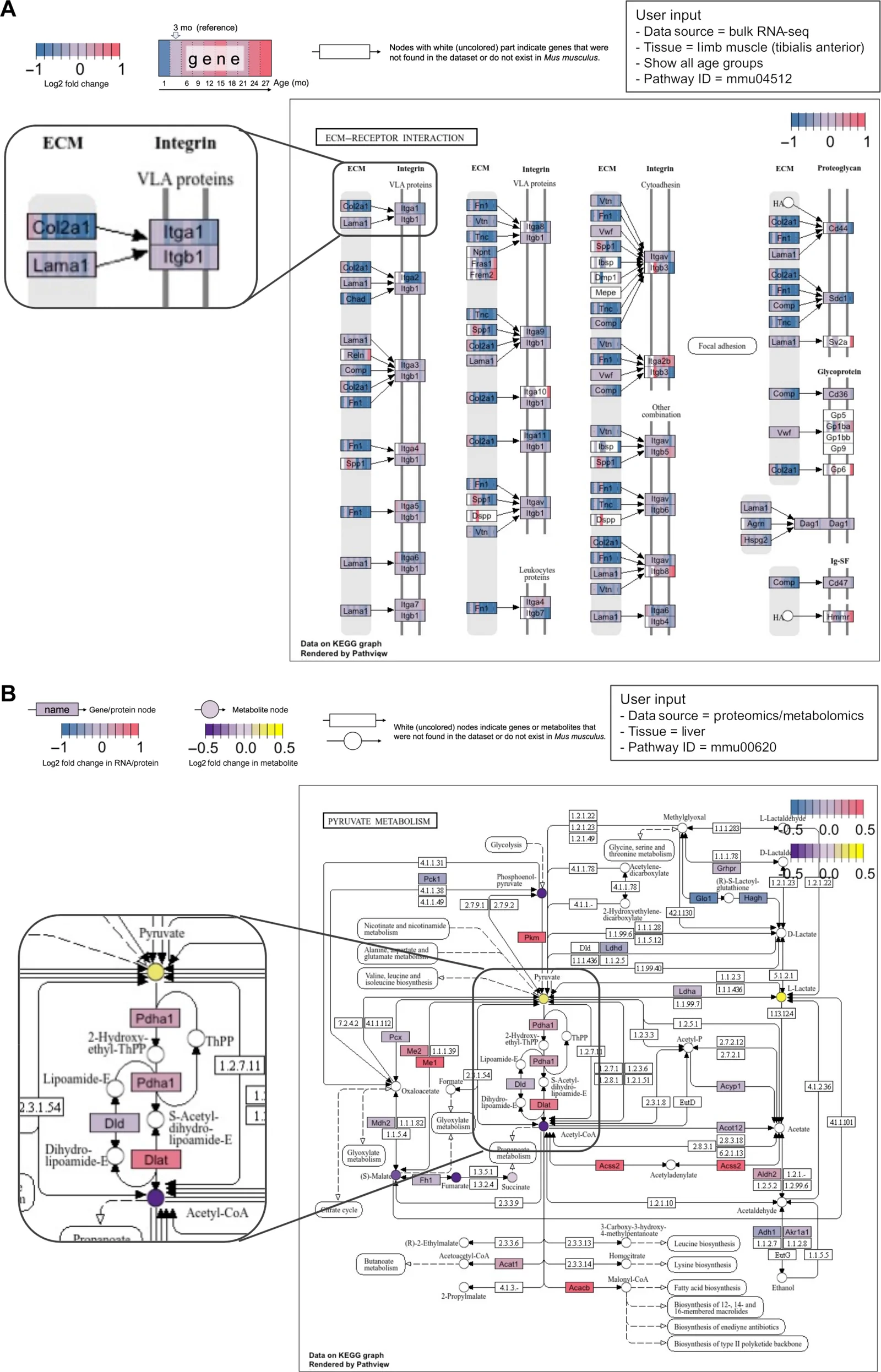
Representative outputs of the pathway mapping module in Shiny AMMOA. (A) Age-associated bulk transcriptomic changes in murine limb muscle (tibialis anterior) mapped onto the “ECM–receptor interaction” pathway (Kyoto Encyclopedia of Genes and Genomes [KEGG] ID: mmu04512). Expression changes across the murine lifespan (1, 3, 6, 9, 12, 15, 18, 21, 24, and 27 mo) are visualized using a heat scale, with the 3-month time point as the reference. Each gene node is divided into columns corresponding to the 10 age points. Missing data are displayed in white, whereas a log2 fold change of 0 is displayed in light gray. (B) Age-associated proteomic (8 mo vs. 18 mo) and metabolomic (3–6 mo vs. >21 mo) changes in murine liver overlaid onto the “Pyruvate metabolism” pathway (KEGG ID: mmu00620). Rectangular nodes (blue-to-red scale) represent protein expression, and circular nodes (purple-to-yellow scale) represent metabolite abundance. The color scales are independently applied to each omics layer. Both the KEGG pathway diagram and graphical legend were generated and downloaded directly from Shiny AMMOA.

Finally, to demonstrate how Shiny AMMOA facilitates cross-omic comparison, age-associated proteomic and metabolomic changes in the “Pyruvate metabolism” pathway (KEGG mmu00620) in liver are visualized in Fig. 5B. Pyruvate metabolism occupies a central position in cellular energy homeostasis, linking glycolysis, gluconeogenesis, and the tricarboxylic acid cycle, and plays a critical role in hepatic metabolic flexibility [31], which is known to decline with aging [32]. Of note, the metabolomic datasets incorporated into Shiny AMMOA do not comprehensively cover all metabolites represented in KEGG pathway maps. As of 2026 September 10, the KEGG compound database (https://www.genome.jp/kegg/compound/) contained 19,632 compound entries, of which 6,688 were linked to pathway maps. In comparison, 300 of the 426 unique metabolites identified in the source article were successfully assigned KEGG identifiers and were therefore available for KEGG-based pathway visualization in Shiny AMMOA (see “Materials and Methods” section). Thus, the metabolites represented in the dataset correspond to approximately 4.5% of all KEGG compound entries linked to pathway maps. This proportion should not, however, be interpreted as inadequate metabolomic coverage. KEGG pathway maps encompass a broad range of chemical compounds, including unstable or/and low-abundance intermediate metabolites that may be difficult to detect using untargeted liquid chromatography–mass spectrometry platforms, as well as compounds that are not produced or are not relevant to mammalian metabolism. Given these considerations, the observed coverage provides a reasonable and informative basis for exploratory pathway visualization, enabling the co-visualization of age-associated changes in metabolic enzymes and measurable metabolites.

## Discussion

In the present report, I developed Shiny AMMOA, a GUI-based, user-friendly analytical application for analysis of predefined murine aging datasets across bulk RNA-seq, proteome, and metabolome. By curating publicly available resources and implementing a modular Shiny-based architecture, Shiny AMMOA enables end-to-end exploratory analyses, including differential expression, enrichment testing, and pathway-level visualization within individual omics layers and across multiple omics layers within a unified interface. Through representative use cases, I illustrated that Shiny AMMOA can recapitulate key findings from original source studies, facilitate cross-omic comparison of pathway enrichment, and enable intuitive visualization of tissue-, pathway-, and modality-specific aging-associated alterations. Such features make Shiny AMMOA particularly valuable for experimental biologists and aging researchers who may not be fully familiar with programming, as it provides them with an opportunity to explore a limited number of curated public datasets, rapidly generate biologically informed hypotheses, and identify pathways or molecular targets for further experimental investigation without requiring custom scripting or specialized computational infrastructure.

While Shiny AMMOA offers user-friendly, interactive analyses of individual omic layer and cross-omic comparison, certain design choices reflect deliberate trade-offs to ensure accessibility and usability for experimental scientists. First, Shiny AMMOA does not support analysis of user-provided datasets beyond the predefined datasets curated and embedded within the software. This design choice arises from a trade-off between workflow simplicity and analytical flexibility. Omics datasets, whether generated from raw experimental measurements or retrieved from public repositories, differ substantially in their levels of preprocessing, annotation, and formatting. Developing a universal software framework capable of accepting all types of datasets would require substantial complexity and could compromise usability for non-specialist users. Furthermore, several GUI-based software tools already support analyses using user-provided datasets, although these tools generally require users to prepare appropriate input data before analysis [9–11]. These tools would serve a complementary purpose by providing flexible analysis of user-generated datasets, whereas Shiny AMMOA focuses on quick exploration of specifically aging-related murine cross-omic resources.

Second, for transcriptomic analyses, Shiny AMMOA currently incorporates only bulk RNA-seq data. Single-cell RNA-seq (scRNA-seq) and single-nucleus RNA-seq (snRNA-seq) can provide greater cell-type-specific resolution. For example, although Shiny AMMOA identified an age-associated alteration in the ECM–receptor interaction pathway in limb muscle (Fig. 5A), bulk RNA-seq cannot distinguish whether such changes reflect alterations in cell-type composition or changes in gene expression within individual cell types. While several murine aging scRNA-seq datasets are publicly available [33], Shiny AMMOA deliberately adopts bulk RNA-seq in accordance with its design philosophy, which may appear counterintuitive. However, single-cell-scale datasets typically involve large data volumes and substantial computational demands, making interactive, GUI-based statistical analyses less practical on entry-level, home-use computers. Bulk RNA-seq therefore represents a pragmatic compromise, providing sufficient insights for initial exploratory analyses while minimizing computational burden.

Beyond these functional trade-offs, Shiny AMMOA also exhibits limitations inherent to the available resources and their licensing requirements. First, 3 omics datasets implemented in Shiny AMMOA were derived from 3 independent studies rather than from the same mice. Therefore, Shiny AMMOA does not support mouse-level pairwise analysis across different omic layers, limiting the types of multi-omic analyses that can be performed. This is relevant because some commonly used multi-omic methods are designed for datasets measured from the same samples or individuals [34]. In addition, the age groups represented in the different omics datasets are not necessarily comparable. For example, the “Old” group in the proteomic dataset corresponds to 18 months of age, whereas the corresponding aged group in the metabolomic dataset includes mice aged ≥21 months although both datasets were derived from C57BL/6J mice. Therefore, cross-omic visualization in Shiny AMMOA should be interpreted with caution, particularly when comparing age-associated changes across omics layers.

Second, because KEGG pathway maps are subject to the KEGG Copyright and Disclaimer, additional KEGG licensing may be required for some users, particularly for nonacademic use. Taking these considerations into account, WikiPathways [35] may provide an alternative open pathway resource. However, WikiPathways is not currently integrated into Shiny AMMOA, primarily because of compatibility considerations with the other analytical software and workflows implemented in the current application.

In conclusion, Shiny AMMOA provides an accessible end-to-end workflow for exploratory analysis of predefined murine aging-associated cross-omic data, providing researchers with rapid access to curated large-scale public datasets through a user-friendly interface. By allowing interactive exploration of preprocessed datasets without requiring coding, the application enables experimental researchers to explore aging-associated datasets and supports hypothesis generation for further experimental investigation.

## Data Availability

All source data underlying this work are available in previously published works (see “Materials and Methods” section). Curated datasets generated during the current work are available at https://doi.org/10.5281/zenodo.22903473. The script workflow for generating application-ready data objects is provided in Fig. S3. All code associated with this work is available at the author’s Zenodo repository (https://doi.org/10.5281/zenodo.22903473). The fully functional local version of Shiny AMMOA is available at https://doi.org/10.5281/zenodo.22903473 with step-by-step setup guide provided at https://github.com/M-Ninomiya-Kanda/Shiny_AMMOA_local. For users who prefer a containerized environment, a Dockerfile and docker-compose.yml are provided in the Zenodo repository above to facilitate environment setup. The software incorporates third-party open-source packages, including clusterProfiler and pathview, which may access KEGG resources. Use of KEGG data and services is subject to the terms and conditions specified by KEGG and is independent of the MIT License of Shiny AMMOA. A detailed user guide describing the analytical workflow and application features is provided as Supplementary User Guide and is also embedded within the application. A lightweight web-based demonstration version for previewing the graphical user interface and analytical workflow is available at https://m-ninomiya-kanda-shiny-ammoa-web.share.connect.posit.cloud/.

## Supplementary Materials

Supplementary 1 Figs. S1 to S7 Tables S1 to S8 Files S1 and S2 Supplementary Appendices 1 and 2
