# Supplementary material for: Shiny Aging Murine Multi-Omic Analyzer: An Interactive Platform for Exploratory Multi-Omic Analysis of Murine Aging
Source: Comput Struct Biotechnol J. 2026 Nov 10;35(1):0245. doi: 10.34133/csbj.0245 (PMC13748626; doi:10.34133/csbj.0245)
Supplement: Supplementary file 1 [file csbj.0245.f1.zip › Supplementary Appendices and Figures_Final.docx]

Supplementary Materials for “Shiny AMMOA: An Interactive Platform for Exploratory Multi-omic Analysis of Murine Aging”

***In this file:***

**Supplementary Figure 1.** Sensitivity of GO (BP) enrichment results to differential expression thresholds.

**Supplementary Figure 2.** Examples of metabolomic differential abundance analysis.

**Supplementary Figure 3.** Workflow for generating application-ready data objects from source data.

**Supplementary Appendix 1.** Reproducibility Analysis of the Original Source Data Articles and Sensitivity Analysis

**Supplementary Figure 4.** Validation of Shiny AMMOA in the proteome layer

**Supplementary Figure 5.** Validation of Shiny AMMOA in the metabolome layer.

Supplementary Figure 6. Sensitivity analysis of metabolomic differential abundance results to OLS model specification

Supplementary Figure 7. Sensitivity of downstream enrichment analysis to OLS model specification

Supplementary Table 7. Analytical settings used for GO enrichment analysis in Keele et al. and Shiny AMMOA.

Supplementary Table 8. The tissue-specific overlap of metabolites passing the default thresholds

**Supplementary Appendix 2.** R Environment Used to Generate the Figures

***Available separately:***

**Supplementary Excel**

**- Supplementary Table 1.** Comparison Table of Three Datasets Used in Shiny AMMOA

**- Supplementary Table 2.** Effective Sample Size for Each Omic Layer, Tissue, Age and Sex Group

**- Supplementary Table 3.** Mapping Results of Compound Common Names to KEGG and HMDB IDs

**- Supplementary Table 4.** Comparison of Shiny AMMOA with Existing GUI-Based Tools

**- Supplementary Table 5.** Comparison Table of Shiny AMMOA local version and web version

**- Supplementary Table 6.** Package Versions for All Major Dependencies Used in the Present Work

**Supplementary File 1:** Userguide_Data_Analysis.pdf

**Supplementary File 2:** Userguide_Source_Data_Description_for_Users.pdf

**Supplementary Figure 1. Sensitivity of GO (BP) enrichment results to differential expression thresholds.**

GO (BP) enrichment analysis of age-associated bulk RNA-seq data from murine spleen using various parameter settings. (a) GSEA. (b–e) ORA using different differential expression thresholds: (b) FDR < 0.05 and |log₂FC| > 2, (c) FDR < 0.05 and |log₂FC| > 1.5, (d) FDR < 0.05 and |log₂FC| > 0.5, and (e) FDR < 0.1 and |log₂FC| > 0. GO (BP), Gene Ontology Biological Process; GSEA, gene set enrichment analysis; ORA, over-representation analysis; FDR, false discovery rate; log₂FC, log₂ fold change.

**Supplementary Figure 2. Examples of metabolomic differential abundance analysis.** Volcano plots showing age-associated changes in metabolites from liver, quadriceps, and soleus samples (3–6 months vs. >21 months). Metabolites that exceed user-defined differential expression thresholds (FDR < 0.1, |log₂ fold change| > 0) are colored. 3-Hydroxybutyric acid is highlighted using the built-in labeling feature of Shiny AMMOA.

**Supplementary Figure 3. Workflow for generating application-ready data objects from source data.**

Supplementary Appendix 1. Reproducibility Analysis of the Original Source Data Articles and Sensitivity Analysis

To quantitatively assess whether Shiny AMMOA can reproduce findings reported in the original source publications, I compared results generated by Shiny AMMOA with those reported in the corresponding studies. Two levels of results were considered for validation: differential expression results and functional enrichment results.

For the proteomic dataset, validation of differential expression results was not performed because Shiny AMMOA uses the same differential expression results generated by the original authors and provided as intermediate data files in FigShare (see Methods). For functional enrichment, I compared Gene Ontology (GO) biological process (BP) enrichment results for age-associated differentially expressed proteins across three tissues (kidney, adipose tissue and liver) published as supplementary table in the original source article and quantified the overlap in significantly enriched GO (BP) terms (Supplementary Figure 4). Among the GO (BP) terms identified by Shiny AMMOA, 76–86% overlapped with those reported in the original study. Conversely, 20–37% of the GO BP terms reported by Keele et al. were also identified by Shiny AMMOA. The lower overlap in the latter direction was likely attributable, at least in part, to differences in the statistical thresholds used for pathway enrichment. Specifically, Keele et al. applied relatively permissive thresholds (padj < 0.1 and q-value < 0.1), whereas Shiny AMMOA used padj < 0.05 and q-value < 0.2, the default settings of clusterProfiler (version 4.20.0). Additional differences may have arisen from differences in the parameters specified for the enrichGO() function, the version of the GO database, and the version of the clusterProfiler or GO.db package. Notably, clusterProfiler internally maps non-ENTREZID input identifiers to ENTREZID for enrichment analysis, although the extent to which this difference contributed to the observed discrepancies was not directly evaluated (Supplementary Table 7).

These results indicate that the enrichment results generated by Shiny AMMOA substantially overlap with those reported in the original study, while also demonstrating that differences in analytical parameters and software/database versions can lead to differences in the specific pathways identified. Accordingly, Shiny AMMOA generally captures a relatively conservative subset of the enrichment results reported in the original study.

**Supplementary Figure 4. Validation of Shiny AMMOA in the proteome layer**

Significantly enriched Gene Ontology (GO) biological process (BP) terms reported by Keele et al. [16] and identified by the corresponding analysis using Shiny AMMOA were compared for kidney, adipose tissue, and liver. The overlap between the two analyses is visualized as Venn diagrams.

Supplementary Table 7. Analytical settings used for GO enrichment analysis in Keele et al. and Shiny AMMOA.

|  | | Keele et al | Shiny AMMOA |
| --- | --- | --- | --- |
| R package/function for EA | | clusterProfiler::enrichGO() | clusterProfiler::enrichGO() |
| Package version | | Not specified | clusterProfiler ver 4.20.0  GO.db ver 3.23.1 |
| DEP cutoff | | FDR < 0.1 & \|log2FC\| > 0 | FDR < 0.1 & \|log2FC\| > 0 |
| enrichGO()  parameters | Input ID type  (keyType) | ENSEMBL | ENTREZID (function default) |
|  | p-value pathway cutoff  (pvalueCutoff) | < 0.1 | < 0.05 |
|  | Q-value pathway cutoff  (qvalueCutoff) | < 0.1 | < 0.2 (function default) |
|  | Ontology  (ont) | All | BP |

For the metabolomic dataset, the original source article did not perform pathway-level enrichment analysis. Therefore, validation was limited to differential expression results by comparing log_2_ fold-change estimates between the source article and Shiny AMMOA. This comparison was particularly relevant because the source publication stated that the authors modeled both the main effect of age and the age × sex interaction, whereas Shiny AMMOA models the main effects of age and sex for consistency with the other omics layers (see Methods). The adjusted log_2_ fold-change estimates for the old-versus-young comparison showed strong concordance across all 15 tissues investigated, with Spearman correlation coefficients (rho) ranging from 0.941 to 1.000 (Supplementary Figure 5).

Notably, the log_2_ fold-change estimates were nearly identical between the two analyses for most tissues, with the exception of colon and pancreas, where relatively greater differences were observed. Because the original publication does not provide sufficient methodological details or the source code required to fully reconstruct its statistical model, the underlying causes of these tissue-specific differences could not be determined. I therefore interpret this comparison primarily as evidence of overall concordance between the two implementations rather than as a direct replication of the original statistical analysis.

**Supplementary Figure 5. Validation of Shiny AMMOA in the metabolome layer.**

Log_2_ fold-change values for metabolite abundance between young (3–6 months) and aged (≥21 months) mice reported by Jankowski et al. [7] were compared with the corresponding values recalculated for Shiny AMMOA across 15 tissues. rho: Spearman correlation coefficient.

The preceding validation compared the age-associated log_2_ fold-change estimates reported in the original study with those generated as application-ready data used in Shiny AMMOA. However, the source article did not provide sufficient methodological details to reconstruct precisely how the published age-associated effects were estimated. Therefore, this comparison alone could not establish the extent to which omitting an age × sex interaction affected the differential-abundance results obtained with Shiny AMMOA.

To assess the sensitivity of downstream results to model specification, I fitted two ordinary least squares (OLS) models using the same processed metabolomic data from individual mice:

Additive model (implemented in Shiny AMMOA): Metabolite abundance ~ Age + Sex

Interaction model (sensitivity analysis): Metabolite abundance ~ Age * Sex

Metabolites were classified as differentially abundant using the default thresholds as implemented in Shiny AMMOA (FDR < 0.1 and |log_2_ fold change| > 0). In the interaction model, male was specified as the reference category; therefore, the age coefficient represented the age-associated difference between aged and young male mice.

Supplementary Figure 6 shows the signed FDR scores, calculated as −sign(log_2_ fold change) × log_10_(FDR), for the two models. Supplementary Table 8 summarizes the tissue-specific overlap of metabolites passing the default thresholds, including counts of metabolites classified as increased or decreased in both models or in only one model. The Jaccard index for the FDR-significant metabolite sets ranged from 0.233 to 0.757 across the 15 tissues, indicating substantial variation in concordance between the two model specifications. Because the two models estimate different age effects, the observed differences in significance calls reflect both the model specification and the estimand, rather than isolating the effect of including an interaction term alone. Thus, the choice of model specification affected the sets of metabolites identified as differentially abundant in an age-associated manner, although the extent of this effect varied across tissues.

Supplementary Figure 6. Sensitivity analysis of metabolomic differential abundance results to OLS model specification

Scatter plots show the signed FDR scores, calculated as −sign(log_2_ fold change) × log_10_(FDR), obtained using the two models for each tissue. The x-axis represents scores from the additive model (implemented in Shiny AMMOA), and the y-axis represents scores from the interaction model (used for sensitivity analysis). Each point represents a metabolite measured in the corresponding tissue and is color-coded according to whether it met the default differential abundance criteria (FDR < 0.1 and |log_2_ fold change| > 0) in both models (black), only the additive model (purple), only the interaction model (green), or neither model (gray). J, Jaccard index.

Supplementary Table 8. The tissue-specific overlap of metabolites passing the default thresholds

| Tissue | Total Measured Metabolites | Increased with Age | | | | | Decreased with Age | | | | Overall Jaccard Index |
| --- | --- | --- | --- | --- | --- | --- | --- | --- | --- | --- | --- |
|  |  | Both | Additive Only | Interaction Only | Jaccard Index | Both | | Additive Only | Interaction Only | Jaccard Index |  |
| Brain | 206 | 3 | 9 | 1 | 0.231 | 6 | | 6 | 10 | 0.273 | 0.257 |
| Colon | 256 | 1 | 0 | 0 | 1.000 | 2 | | 3 | 1 | 0.333 | 0.429 |
| Diaphragm | 225 | 6 | 8 | 4 | 0.333 | 31 | | 25 | 3 | 0.525 | 0.481 |
| Eye | 228 | 48 | 21 | 2 | 0.676 | 51 | | 14 | 9 | 0.689 | 0.694 |
| Heart | 215 | 11 | 7 | 5 | 0.478 | 24 | | 23 | 4 | 0.471 | 0.473 |
| Jejunum | 298 | 3 | 6 | 1 | 0.300 | 4 | | 14 | 2 | 0.200 | 0.233 |
| Kidney | 228 | 13 | 11 | 6 | 0.433 | 127 | | 8 | 20 | 0.819 | 0.757 |
| Liver | 225 | 24 | 15 | 10 | 0.490 | 37 | | 14 | 27 | 0.474 | 0.480 |
| Lung | 201 | 39 | 25 | 5 | 0.565 | 16 | | 9 | 7 | 0.500 | 0.545 |
| Pancreas | 232 | 6 | 12 | 2 | 0.300 | 23 | | 42 | 5 | 0.329 | 0.322 |
| Quadriceps | 180 | 5 | 3 | 7 | 0.333 | 97 | | 1 | 42 | 0.693 | 0.658 |
| Serum | 170 | 7 | 2 | 7 | 0.438 | 35 | | 17 | 3 | 0.636 | 0.592 |
| Skin(Ear) | 344 | 7 | 21 | 2 | 0.233 | 94 | | 42 | 26 | 0.580 | 0.526 |
| Soleus | 221 | 9 | 8 | 9 | 0.346 | 22 | | 14 | 1 | 0.595 | 0.492 |
| Spleen | 237 | 15 | 25 | 3 | 0.349 | 12 | | 6 | 10 | 0.429 | 0.380 |

To assess the potential downstream consequences, KEGG-based ORA was compared between the two models for the pancreas and colon (Supplementary Figure 7). In the pancreas, the adjusted p-value for “Cysteine and methionine metabolism” (KEGG pathway ID: mmu00270) was 0.725 under the additive model and 0.061 under the interaction model, although both models identified six FDR-significant metabolites mapped to this pathway. Thus, the adjusted p-value was substantially lower under the interaction model, although the pathway did not reach the significance threshold under either model. In the colon, no KEGG pathways were significantly enriched under either model. This is potentially due to the very limited number of metabolites available for enrichment analysis in colon; only three and one FDR-significant metabolites could be mapped to KEGG IDs under the additive and interaction models, respectively.

Supplementary Figure 7. Sensitivity of downstream enrichment analysis to OLS model specification

Differentially abundant metabolites were identified using two model specifications: the additive model (implemented in Shiny AMMOA) and the interaction model (used for sensitivity analysis). The resulting metabolite sets were subjected to KEGG pathway enrichment analysis using the same over-representation analysis (ORA) procedure as implemented in Shiny AMMOA. (a) Pancreas, additive model. (b) Pancreas, interaction model. (c) Colon, additive model. (d) Colon, interaction model. ORA, over-representation analysis.

Taken together, this sensitivity analysis shows that the inclusion of an age × sex interaction can alter the metabolites identified as differentially abundant and, in some cases, the downstream ORA results, including pathway-wise adjusted p-values, although these changes may not necessarily alter pathway-significance classifications. Shiny AMMOA currently uses an additive model with age and sex as main effects and does not estimate sex-specific age effects or age × sex interactions. To help users interpret the results appropriately, this model specification is explicitly described in the User Guide (Supplementary File 1).

Finally, I considered validation of the transcriptomic layer. However, I was unable to identify publicly available materials that could be quantitatively compared with Shiny AMMOA for both differential expression and pathway-level enrichment analyses. The Tabula Muris Senis data visualizer (https://twc-stanford.shinyapps.io/maca/, last accessed September 18, 2026) provides volcano plot visualization; however, the underlying differential expression tables are displayed only and are not available for download. For pathway-level enrichment, the source article presents cross-tissue enrichment analyses based on differentially expressed genes more than one tissues and gene clustering followed by over-representation analysis, but does not report conventional tissue-specific enrichment analyses, including in the extended materials. Therefore, a direct quantitative validation of the transcriptomic enrichment results generated by Shiny AMMOA was not feasible based on the publicly available materials.

Supplementary Appendix 2. R Environment Used to Generate the Figures

Please note that the R session information provided below corresponds to the environment used to run Shiny AMMOA and generate the main figures. Data preprocessing to generate application-ready, curated data objects, including differential expression analysis using DESeq2, was performed in separate R environments. Package information for data preprocessing is provided in the corresponding Methods section and Supplementary Table 6.

R version 4.6.0 (2026-04-24)

Platform: aarch64-apple-darwin23

Running under: macOS Tahoe 26.6.2

Matrix products: default

BLAS: /System/Library/Frameworks/Accelerate.framework/Versions/A/Frameworks/vecLib.framework/Versions/A/libBLAS.dylib

LAPACK: /Library/Frameworks/R.framework/Versions/4.6/Resources/lib/libRlapack.dylib; LAPACK version 3.12.1

locale:

[1] en_US.UTF-8/en_US.UTF-8/en_US.UTF-8/C/en_US.UTF-8/en_US.UTF-8

time zone: America/Los_Angeles

tzcode source: internal

attached base packages:

[1] stats	graphics grDevices utils	datasets methods	base

other attached packages:

[1] lubridate_1.9.5	forcats_1.0.1	stringr_1.6.0	dplyr_1.2.1

[5] purrr_1.2.2	readr_2.2.0	tidyr_1.3.2	tibble_3.3.1

[9] ggplot2_4.0.3	tidyverse_2.0.0	pathview_1.52.0	clusterProfiler_4.20.0

[13] png_0.1-9	ggiraph_0.9.6	viridis_0.6.5	viridisLite_0.4.3

[17] RColorBrewer_1.1-3	XML_3.99-0.23	htmltools_0.5.9	httr_1.4.8

[21] waiter_0.2.5.1	reactable_0.4.5	bsplus_0.1.5	bslib_0.11.0

[25] shiny_1.13.0

loaded via a namespace (and not attached):

[1] rstudioapi_0.19.0	jsonlite_2.0.0	tidydr_0.0.6	magrittr_2.0.5

[5] ggtangle_0.1.2	farver_2.1.2	ragg_1.5.2	fs_2.1.0

[9] vctrs_0.7.3	config_0.3.2	memoise_2.0.1	RCurl_1.98-1.19

[13] ggtree_4.2.0	shinycustomloader_0.9.0	gridGraphics_0.5-1	sass_0.4.10

[17] fontawesome_0.5.3	htmlwidgets_1.6.4	plyr_1.8.9	httr2_1.2.2

[21] cachem_1.1.0	igraph_2.3.2	mime_0.13	lifecycle_1.0.5

[25] pkgconfig_2.0.3	R6_2.6.1	fastmap_1.2.0	gson_0.1.0

[29] digest_0.6.39	aplot_0.2.9	enrichplot_1.32.0	ggnewscale_0.5.2

[33] patchwork_1.3.2	AnnotationDbi_1.74.0	S4Vectors_0.50.1	aisdk_1.4.12

[37] textshaping_1.0.5	crosstalk_1.2.2	RSQLite_3.53.2	MicrobiomeProfiler_1.18.1

[41] reactR_0.6.1	org.Hs.eg.db_3.23.1	labeling_0.4.3	org.Mm.eg.db_3.23.0

[45] timechange_0.4.0	polyclip_1.10-7	compiler_4.6.0	attempt_0.3.1

[49] bit64_4.8.2	fontquiver_0.2.1	withr_3.0.2	S7_0.2.2

[53] DBI_1.3.0	ggforce_0.5.0	MASS_7.3-65	rappdirs_0.3.4

[57] tools_4.6.0	otel_0.2.0	ape_5.8-1	scatterpie_0.2.6

[61] httpuv_1.6.17	glue_1.8.1	callr_3.8.0	nlme_3.1-169

[65] GOSemSim_2.38.0	promises_1.5.0	grid_4.6.0	rsconnect_1.11.0

[69] cluster_2.1.8.2	reshape2_1.4.5	generics_0.1.4	gtable_0.3.6

[73] tzdb_0.5.0	hms_1.1.4	XVector_0.52.0	BiocGenerics_0.58.1

[77] ggrepel_0.9.8	pillar_1.11.1	yulab.utils_0.2.4	later_1.4.8

[81] splines_4.6.0	tweenr_2.0.3	treeio_1.36.1	lattice_0.22-9

[85] bit_4.6.0	tidyselect_1.2.1	fontLiberation_0.1.0	GO.db_3.23.1

[89] Biostrings_2.80.1	fontBitstreamVera_0.1.1	gridExtra_2.3	IRanges_2.46.0

[93] Seqinfo_1.2.0	stats4_4.6.0	Biobase_2.72.0	DT_0.34.0

[97] KEGGgraph_1.72.0	stringi_1.8.7	lazyeval_0.2.3	ggfun_0.2.0

[101] yaml_2.3.12	shinyWidgets_0.9.1	gdtools_0.5.1	qvalue_2.44.0

[105] Rgraphviz_2.56.0	graph_1.90.0	ggplotify_0.1.3	cli_3.6.6

[109] xtable_1.8-8	systemfonts_1.3.2	processx_3.9.0	jquerylib_0.1.4

[113] golem_0.5.1	dichromat_2.0-0.1	Rcpp_1.1.1-1.1	parallel_4.6.0

[117] blob_1.3.0	DOSE_4.6.0	bitops_1.0-9	tidytree_0.4.7

[121] enrichit_0.1.5	scales_1.4.0	crayon_1.5.3	rlang_1.2.0

[125] KEGGREST_1.52.0
