# Supplementary material for: Shiny Aging Murine Multi-Omic Analyzer: An Interactive Platform for Exploratory Multi-Omic Analysis of Murine Aging
Source: Comput Struct Biotechnol J. 2026 Nov 10;35(1):0245. doi: 10.34133/csbj.0245 (PMC13748626; doi:10.34133/csbj.0245)
Supplement: Supplementary file 1 [file csbj.0245.f1.zip › Userguide_Data_Analysis.pdf]

Shiny AMMOA User Guide

Last Modified 2026/09/21

- Web vs Local Implementation: Features and Limitations
- KEGG License Note
- bulk RNA / Proteome Page
  - Side Panel: User-defined Parameters
    - Tissue
    - Enrichment Analysis Algorithm
    - Pathway Database
    - Age Groups
    - DEG/DEP Threshold
    - DEs Used for Enrichment Analysis
  - Main Tab: Volcano Plot
    - Volcano Plot
    - Result Table
  - Main Tab: Pathway Enrichment
    - Dot Plot
    - All Enriched Biological Terms
- Metabolome Page
  - Side Panel: User-defined Parameters
    - Tissue
    - Enrichment Analysis Algorithm
    - Database
    - DE Threshold
    - DEs Used for Enrichment Analysis
    - Background Metabolites
  - Main Tab: Metabolite Volcano Plot
    - Volcano Plot
    - Result Table
  - Main Tab: Metabolite Pathway Enrichment
    - Dot Plot
    - All Biological Terms
- Pathway Mapping
  - What Pathway Mapping can Do
  - Side Panel: User-defined Parameters
    - Source of Gene/Protein Nodes
    - Source of Metabolite Nodes
    - KEGG Pathway
- Statistical Note
  - What is Covariate Adjustment?

Web vs Local Implementation: Features and Limitations

This application is available in two forms: the **Web version** and the **Local version**. Here are the key differences and limitations.

Web Version (Lightweight)
The app runs on the Posit Connect Cloud web server.

Pros:

- No need to install any software or R packages.

Cons:
Due to compliance with external database terms and conditions, as well as server-side resource limitations (e.g., RAM and CPU constraints), some features are restricted. For example:

- KEGG pathway diagrams cannot be displayed due to licensing restrictions database. See next section for details.
- Some parameters that can be adjusted in the local version are fixed in the web version (e.g., bulk RNA-seq group composition for differential expression analysis and differential expression thresholds).

Detailed limitations are explained in each section below.
Additionally, because of resource constraints, the app may not be available when multiple users access the website simultaneously or when multiple analysis requests are submitted in a short period. Please note that the web version is intended primarily as a showcase of the user interface and therefore is not suitable for intensive analyses.

Local Version (Full-featured)
The app runs on your own computer.

Pros:
Full-features of the app are available.

Cons:

- Users must download and run the source code from the author's GitHub.
- You must also install R/RStudio and all required CRAN and Bioconductor packages if not already installed. Installation instructions are provided on the GitHub page.

KEGG License Note

Shiny AMMOA is implemented with consideration of the licensing conditions specified in the KEGG Copyright and Disclaimer (last accessed: 2026-09-17).
The licensing of the Shiny AMMOA software is independent of the licensing conditions applicable to KEGG content. In particular, non-academic users should obtain the appropriate KEGG license before using KEGG data.
Moreover, the web version of Shiny AMMOA does not display KEGG pathway to avoid providing KEGG-derived pathway content as part of a hosted web service.

bulk RNA / Proteome Page

Side Panel: User-defined Parameters

Tissue

Choose the tissue to analyze. 17 tissues are available for bulk RNA-seq and 10 for proteomics. For details, please refer to the **Source Data** tab.

Enrichment Analysis Algorithm

Choose the algorithm applied for enrichment analysis.

- **ORA** (Over-Representation Analysis)
  This is a conventional enrichment analysis algorithm that evaluates whether a set of differentially expressed genes or proteins (DEGs/DEPs), defined using the user-specified thresholds, is enriched in particular biological pathways.
- **GSEA** (Gene Set Enrichment Analysis)
  GSEA evaluates pathway enrichment based on the overall distribution of gene or protein-level changes, without defining DEGs/DEPs using a pre-defined threshold.

For more technical details, please refer to the relevant literature, such as this review article.

Pathway Database

This option specifies the biological pathway database to be queried for enrichment analysis.

- **KEGG**: Kyoto Encyclopedia of Genes and Genomes
  https://www.kegg.jp/kegg/pathway.html
- **GO(BP)**: Gene Ontology (Biological Process)
- **GO(MF)**: Gene Ontology (Molecular Function)
- **GO(CC)**: Gene Ontology (Cellular Component)
  https://geneontology.org/docs/ontology-documentation/

Age Groups

Note: This parameter is adjustable only for bulk RNA-seq data.
Select the age groups used to detect differential expression.

- **across all ages** (available for bulk RNA-seq only)
  Uses all age groups (1, 3, 6, 9, 12, 15, 18, 21, 24, 27 months) in a linear model.

Pros: Higher statistical power due to larger sample size.
Cons: May fail to detect non-linear patterns, such as U-shaped, bell-shaped, or saturation effects.

- **two age groups comparison**
  Performs comparison between two selected age groups.
  Select the age of **Young** and **Old** groups from the drop-down list below. For proteins, only 8-month vs 18-month is available; for bulk RNA-seq, any pair among 1, 3, 6, 9, 12, 15, 18, 21, 24, 27 months can be selected in the local version.
  Note: In the web version bulk RNA-seq two-group comparison is restricted to 3-month vs 18-month.

DEG/DEP Threshold

These parameters define the criteria used to identify differentially expressed genes/proteins (DEGs/DEPs) for ORA.

*Note: This box appears only when **ORA** is selected as the enrichment analysis algorithm. GSEA does not require a DE threshold, and therefore this box is collapsed when GSEA is selected.*

- **FDR (adjusted p)**
  The false discovery rate (FDR), controlled using the Benjamini–Hochberg procedure.

- **Log2 Fold Change**
  The covariate adjusted log2-transformed fold change in gene/protein expression.

*Note: If you choose the option **across all ages** for **Age Groups**, log2FC values are scaled per 12 months of age. In this case, a log2FC = 1 means the average expression of the gene is estimated to double every 12 months.*

DEs Used for Enrichment Analysis

This option determines the settings for ORA.

- **both**: both differentially up- and down- regulated genes/proteins with age will be used for ORA.
- **up-regulated**: only differentially up-regulated genes/proteins with age will be used for ORA.
- **down-regulated**: only differentially down-regulated genes/proteins with age will be used for ORA.

Main Tab: Volcano Plot

Volcano Plot

Displays the volcano plot.

- Orange and blue dots represent up- and down-regulated genes/proteins with age, respectively, based on the user-defined thresholds (see previous DEG/DEP Threshold section). When GSEA is selected as the enrichment analysis algorithm, the default DE threshold is used for visualization only to color-code genes/proteins.

- The x-axis shows covariate-adjusted log2 fold change, not the crude (raw) fold change. The covariates included in the model vary depending on the selected data source. For details, see the Statistical Note section.
- Hover over each dot to view the gene/protein symbol name.
- Click the **Download Plot** button to save a plot with .png format.

Tip: Use "Labels to Show" window box to show and download the image with gene/protein name labeled.

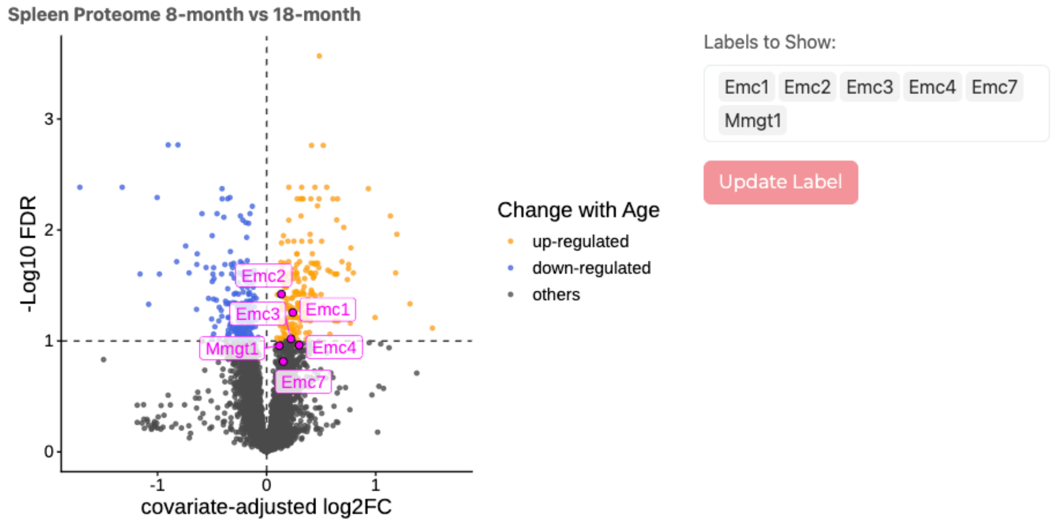

Result Table

Shows summary statistics include of all individual genes/proteins analyzed.

Main Tab: Pathway Enrichment

Dot Plot

- Displays significantly enriched biological pathways (adjusted p-value < 0.05).
- If more than 10 significant pathways are identified, they are grouped into 5 clusters based on the similarity of their component genes/proteins (clusters 1–5).
- If more than 25 significant pathways are identified, only the top 5 pathways from each cluster are displayed.

All Enriched Biological Terms

- A full table of all significant pathways, including those not shown in the Dot Plot.
- Includes details for each pathway, such as pathway ID, statistical values, and differentially expressed pathway components.

Metabolome Page

Side Panel: User-defined Parameters

Tissue

Choose the tissuse to analyze. 15 tissues are available. For details, please refer to the **Source Data** tab.

Enrichment Analysis Algorithm

For what ORA means, please see the related section in **bulk RNA / Proteome** guide.
For metabolomic data, only ORA is supported and GSEA is not available given the nature of the dataset such as limited number of the analytes.

Database

This option specifies the biological pathway database to be queried for enrichment analysis.

- **KEGG**: Kyoto Encyclopedia of Genes and Genomes
  https://www.kegg.jp/kegg/pathway.html
- **SMPDB**: The Small Molecule Pathway Database
  https://smpdb.ca/

DE Threshold

These parameters define the criteria used to identify differentially regulated metabolites.

- **FDR (adjusted p)**
  The false discovery rate (FDR), controlled using the Benjamini–Hochberg procedure.
- **Log2 Fold Change**
  The covariate adjusted log2-transformed fold change.

DEs Used for Enrichment Analysis

This option determines the settings for ORA.

- **both**: both differentially up- and down- regulated metabolites with age will be used for ORA.
- **up-regulated**: only differentially up-regulated metabolites with age will be used for ORA.
- **down-regulated**: only differentially down-regulated metabolites with age will be used for ORA.

Background Metabolites

This parameter specifies the background metabolite set used for enrichment analysis.
In most types of enrichment analysis (e.g., transcriptomics), the background should be limited to the measured biomolecules only (the default choice). This is important for controling false discoveries.

However, in datasets where the number of measured biomolecules is relatively small (such as metabolomic datasets) using only the measured biomolecules as the background can make it difficult to detect altered pathways. In such cases, the option “All metabolites registered in the database” may be used for exploratory analysis. Please note that this option may discover more pathways, but it also increases the risk of false positives.

Main Tab: Metabolite Volcano Plot

Volcano Plot

Displays the volcano plot.
This works in the same way as the volcano plot from bulk RNA/protein analysis. Please see the section above.

Result Table

Shows summary statistics include of all individual metabolites analyzed.

Main Tab: Metabolite Pathway Enrichment

Dot Plot

Displays discovered biological pathways.

All Biological Terms

- A full table of all investigated pathways.
- Includes details for each pathway, such as pathway ID, statistical values, and differentially expressed pathway components.

Pathway Mapping

NOTE: This featre is disabled in the Web version due to KEGG license restrictions (see the previous section).

What Pathway Mapping can Do

This page uses pathview to visualize age-associated change in gene/protein/metabolite expression.
Pathview is an R/Bioconductor package that maps your expression data onto KEGG pathway diagrams using a heat-color scale, allowing you to easily see where up- and down-regulated molecules appear within each pathway.
In this app, rectangular nodes represent gene/proteins, while small circular nodes represent metabolites.

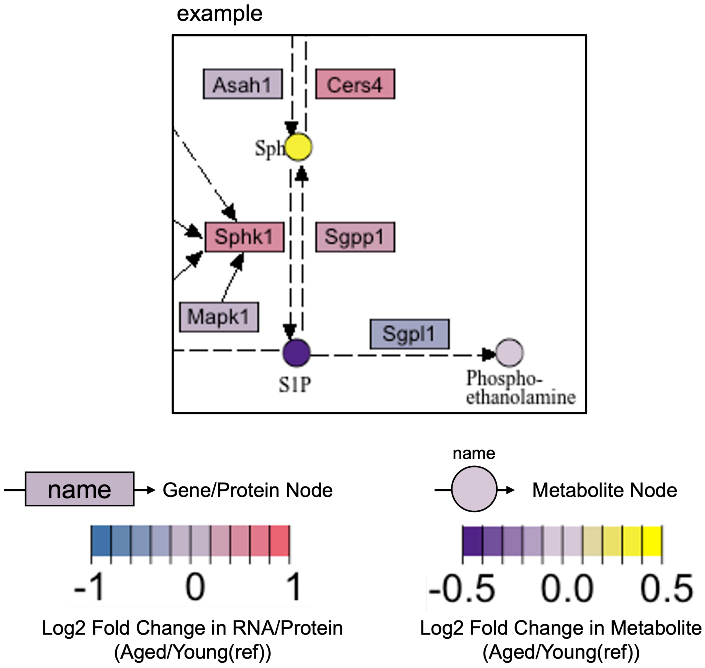

Side Panel: User-defined Parameters

Source of Gene/Protein Nodes

Select the data type and tissue used to display rectangular nodes: bulk RNA-seq (Schaum et al. 2020), proteomics (Keeke et al. 2023), or "not show."
For proteomic data, differential expression is based on an 8-month vs 18-month comparison. For bulk RNA-seq, you must specify the age groups for comparison from the following options: 1, 3, 6, 9, 12, 15, 18, 21, 24, and 27 months. "across all ages" option is only available for bulk RNA-seq analysis (see schematic below).

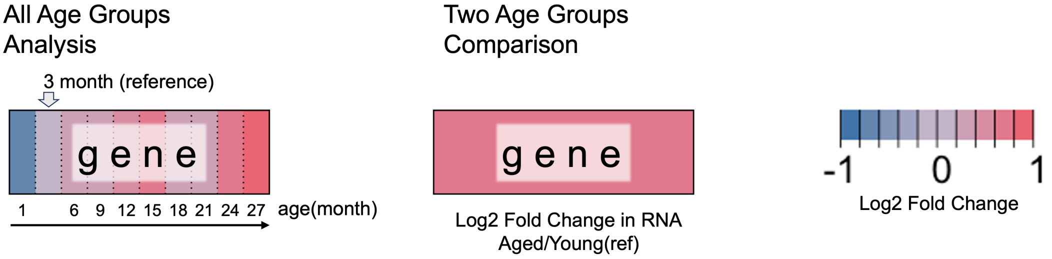

Source of Metabolite Nodes

Select the data type and tissue used to display small circular nodes from metabolomic data (Jankowski et al. 2025), or "not show."

KEGG Pathway

Select the KEGG pathway to display.

- *Tips: The dropdown list is searchable. Type keywords to quickly find a pathway instead of scrolling through the full list.*
- *Note: Some KEGG pathway maps (e.g., mmu01100 and mmu00514 do not contain gene/protein (rectangular) nodes or metabolite (circular) nodes, or contain neither. In such cases, a non-colored pathway image will be shown along with a message.*

Statistical Note

What is Covariate Adjustment?

Because tissue samples in each source dataset were obtained from mice that differed not only in age but also in other factors (typically sex), all analyses were performed with covariate adjustment. The covariate(s) included for each dataset type were as follows:

- **bulk RNA-seq**: Sex was included as a covariate using the built-in pipeline of the DESeq2 package.
- **Proteome**: Sex and analytical batch were included as covariates. For details, see the original article.
- **Metabolome**: Sex was included as a covariate.
