# Supplementary material for: Shiny Aging Murine Multi-Omic Analyzer: An Interactive Platform for Exploratory Multi-Omic Analysis of Murine Aging
Source: Comput Struct Biotechnol J. 2026 Nov 10;35(1):0245. doi: 10.34133/csbj.0245 (PMC13748626; doi:10.34133/csbj.0245)
Supplement: Supplementary file 1 [file csbj.0245.f1.zip › Userguide_Source_Data_Description_for_Users.pdf]

Source Data Used in this App

- bulk RNAseq Dataset
  - General Information
  - Sample Information
  - Original Data Repository
- Proteomic Dataset
  - General Information
  - Sample Information
  - Original Data Repository
- Metabolomic Dataset
  - General Information
  - Sample Information
  - Original Data Source

Below are brief descriptions of the source datasets used in this app. For more details, please refer to the linked original paper.

bulk RNAseq Dataset

General Information

Tabula Muris Senis (bulk Dataset)
https://tabula-muris-senis.sf.czbiohub.org

Description
Tabula Muris Senis is a comprehensive transcriptomic cell atlas of mice covering a wide range of ages. The atlas includes both single-cell RNA-seq and bulk RNA-seq data. In this app, only bulk RNA-seq data are used.

Link to the Original Paper
Schaum N, Lehallier B, Hahn O, et al. Ageing hallmarks exhibit organ-specific temporal signatures. Nature. 2020;583(7817):596-602.
doi:10.1038/s41586-020-2499-y

Sample Information

Mouse Strain
C57BL/6JN

Age/Sex Group Composition

- male mice (n = 4; aged 1, 3, 6, 9, 12, 15, 18, 21, 24 and 27 months)
- female mice (n = 2; aged 1, 3, 6, 9, 12, 15, 18 and 21 months)

Note: The numbers above indicate the number of mice; actual sample counts per tissue may be lower due to quality filtering.

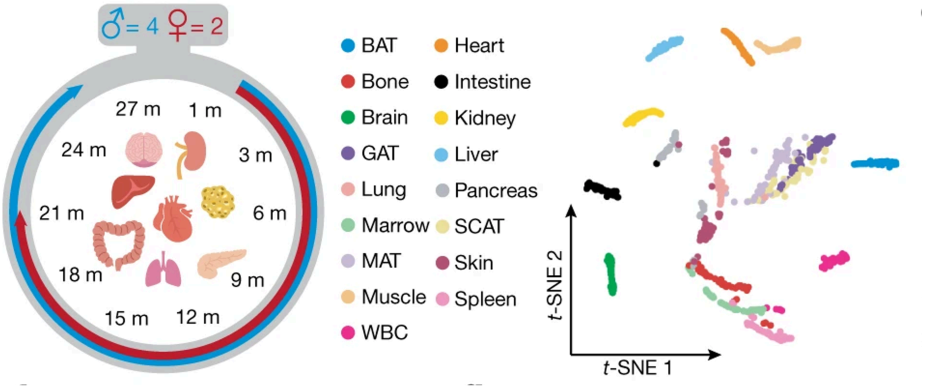

(Schaum et al. 2020)

Tissue
Samples from 17 tissues are available:

- **BAT**: brown adipose tissue (interscapular depot)
- **Bone** (femurs and tibiae)
- **Brain** (hemibrain)
- **GAT**: gonadal adipose tissue (inguinal depot)
- **Heart**
- **Kidney**
- **Limb Muscle** (tibialis anterior)
- **Liver**
- **Lung**
- **Marrow**: bone marrow
- **MAT**: mesenteric adipose tissue
- **Pancreas**
- **SCAT**: subcutaneous adipose tissue (posterior depot)
- **Skin** (dorsal)
- **Small Intestine** (duodenum)
- **Spleen**
- **WBC**: white blood cells (buffy coat)

Measurement Platform
Illumina NovaSeq 6000 (Mus musculus)

Original Data Repository

Gene Expression Omnibus (GSE132040)

Proteomic Dataset

General Information

Description
Proteomic data were retrieved from the paper by Keele et al. (2023). In this study, the authors performed discovery-based proteomics using tandem mass tags (TMTs) in 10 tissues from 20 C57BL/6J mice, including both sexes and two age groups (8 and 18 months).

Link to the Original Paper
Keele GR, Zhang JG, Szpyt J, et al. Global and tissue-specific aging effects on murine proteomes. Cell Rep. 2023;42(7):112715.
doi:10.1016/j.celrep.2023.112715

Sample Information

Mouse Strain
C57BL/6J

Age/Sex Group Composition

- 8-month male (N=5)
- 8-month female (N=5)
- 18-month male (N=5)
- 18-month female (N=5)

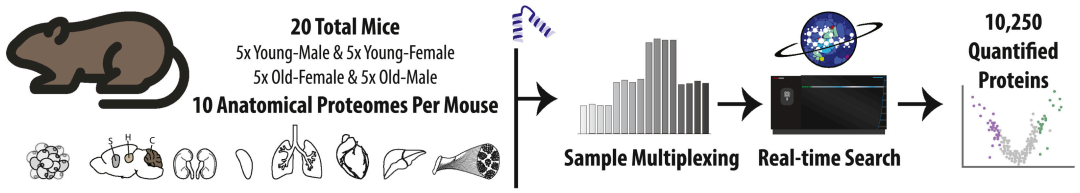

(Keele et al. 2023)

Tissue
10 tissues were analyzed:

- **Kidney**
- **Lung**
- **Liver**
- **Heart**
- **Skeletal Muscle** (quadriceps)
- **Fat** (inguinal adipose)
- **Hippocampus**
- **Cerebellum**
- **Striatum**

Measurement Platform
Tandem mass tags (TMTs)

Original Data Repository

Fig Share (DOI link)

Metabolomic Dataset

General Information

Description
Metabolomic data were retrieved from the paper by Jankowski et al. (2025). In this study, the authors performed both metabolomics and stable isotope tracing on young (3–6 months) and aged (+21 months) C57BL/6J mice. In this app, only the metabolomic data are used.

Link to the Original Paper
Jankowski CSR, Samarah LZ, MacArthur MR, et al. Aged mice exhibit widespread metabolic changes but preserved major fluxes. Cell Metab. 2025;37(11):2280-2294.e4.
doi:10.1016/j.cmet.2025.09.009

Sample Information

Mouse Strain
C57BL/6J

Age/Sex Group Composition
Each group (young male, young female, aged male, aged female) consisted of 8–10 samples per tissue (Approximately 70% of tissue–sex–age groups had N = 10).

Tissue
15 tissues (including serum) were analyzed:

- **Serum**
- **Brain**
- **Eye**
- **Diaphragm**
- **Heart**
- **Jejunum**
- **Kidney**
- **Liver**
- **Lung**
- **Pancreas**
- **Spleen**
- **Colon**
- **Quadriceps**
- **Soleus**
- **Skin (Ear)**

Measurement Platform
Hydrophilic interaction chromatography (HILIC) coupled with a quadrupole-orbitrap mass spectrometer (QExactive Plus, Exploris 240, or Exploris 480)

Original Data Source

Raw data were obtained from the Supplementary Table (Table S6) of the original publication. Individual metabolite data are also available at the R/Shiny app developed by the authors: https://agingmet.princeton.edu/ (last accessed 2025-12-08)
